# A Novel Self-Competitive Fishing Primer qPCR Approach for Efficient POLE Mutation Detection in Endometrial Cancer Molecular Classification

**DOI:** 10.3390/cimb48030257

**Published:** 2026-02-27

**Authors:** Chao-Chih Wu, Yu-Chia Hsiao, Zi-Yu Lin, Pai-Hsuan Chiu, Chih-Long Chang

**Affiliations:** 1 Department of Medical Research, MacKay Memorial Hospital, Taipei City 104217, Taiwan; 2 Institute of Biomedical Sciences, MacKay Medical University, New Taipei City 252005, Taiwan; 3 Department of General Education (Sanzhi Campus), Mackay Junior College of Medicine, Nursing and Management, New Taipei City 252005, Taiwan; 4 Department of Obstetrics and Gynecology, Mackay Memorial Hospital, Taipei City 104217, Taiwan; 5 Department of Medicine, MacKay Medical University, New Taipei City 252005, Taiwan

**Keywords:** POLE mutation, endometrial cancer, qPCR, mutation detection, exonuclease domain mutations (EDMs), next generation sequence (NGS)

## Abstract

This study developed and validated a Self-competitive Fishing (SCF) primer qPCR system as a rapid, cost-effective alternative to next-generation sequencing (NGS) for detecting POLE exonuclease domain mutations (EDMs) in endometrial cancer. The system detects 11 pathogenic POLE EDMs using SuperSelective primers combined with wild-type-blocking oligonucleotides that prevent amplification of wild-type DNA, thereby enhancing mutant DNA detection. The validation process involved comparing specificity using genomic DNA from tumors with known POLE mutations identified by NGS. Sensitivity testing used POLE-mutated DNA diluted in wild-type DNA, while precision was confirmed by analyzing 86 endometrial cancer samples against NGS results. The SCF qPCR system demonstrated superior specificity compared to the original SuperSelective primer-based qPCR, achieving 1% mutation-detection sensitivity across various mutation points. Importantly, results from all endometrial cancer cases showed complete concordance with NGS analysis for the 11 pathogenic POLE-EDM points tested. This cost-effective and efficient SCF primer qPCR system provides an accessible method for routine molecular classification of endometrial cancer in clinical settings, offering a practical alternative to NGS for detecting pathogenic POLE mutations and supporting clinical decision-making.

## 1. Introduction

Several molecular classification systems for endometrial cancer exist today, including the TCGA, ProMisE, and ESMO-ESGO-ESTRO classifications [1,2,3]. Each classification shares significant similarities. According to these classifications, several characteristics significantly impact the prognoses of endometrial cancer: POLE and MSI groups, known for high mutation rates and favorable prognoses; p53 abnormal group, which contains p53 mutations and is associated with a poor prognosis; and high copy number/serous cancer type, characterized by chromosomal instability and a poor prognosis. In clinical practice, these classifications are typically confirmed through immunostaining of tumor tissues [4,5,6,7]. Immunostaining is used to quickly screen for MMR (Mismatch Repair) deletions and p53 mutations, specifically targeting MLH1, MSH2, MSH6, and PMS2 proteins. Loss of staining for any of these proteins indicates an abnormal result, suggesting MMR deletion deficiencies or p53 mutations. The concurrent loss of MLH1 and PMS2, or of MSH2 and MSH6, suggests MMR loss and is indicative of MSI. However, POLE gene mutations cannot be detected by immunostaining. To identify these mutations, Next-Generation Sequencing (NGS) is generally required [8]. This technique detects mutations in the POLE gene in tumors. It is expensive, requires a large sample size, and takes longer to produce the report. As a result, many patients have not had this testing, leading to incomplete molecular classifications. Therefore, the development of a more straightforward, cost-effective, non-NGS-based method for detecting POLE mutations would significantly aid clinical practice by enabling rapid, affordable, large-scale screening.

Current non-NGS mutation-detection methods present significant barriers to routine POLE gene mutation screening in clinical practice. While traditional Sanger sequencing is accessible, it frequently yields false-negative results due to limited sensitivity in highly heterogeneous tumor specimens [9,10]. To enhance sensitivity, wild-type blocking-PCR (WTB-PCR) utilizes specific oligonucleotides to block non-mutant sequences [11,12,13]; however, this approach becomes expensive due to the requirement for additional oligonucleotides, particularly for multi-site detection [14,15]. Simpler alternatives, such as the amplification-refractory mutation system (ARMS), use 3′ mismatches for differentiation but often fail to provide sufficient allelic discrimination [16,17]. While Dual Priming Oligonucleotide (DPO) and SuperSelective primers improved upon this by using anchor and “foot” regions to create melting temperature (Tm) differences [18,19,20], they require labor-intensive, iterative optimization of bridge and foot lengths. Collectively, these technical and economic constraints have hindered the development of a scalable, practical POLE mutation detection system suitable for routine clinical usage.

The present study introduces a novel design, called Self-competitive Fishing (SCF) primer systems, that combines WTB-PCR and SuperSelective primer-based PCR into a single primer, enhancing sensitivity and accuracy in detecting single-point mutations, specifically targeting pathological mutations in the POLE gene exonuclease region. Unlike SuperSelective primers, the bridging region in this design is complementary to the wild-type sequence at the mutation site, making wild-type amplification more difficult and improving detection specificity while reducing false positives. This approach eliminates the need for additional oligonucleotides to block wild-type sequences. Additionally, the combinatorial nucleotides of these primers do not require special modifications, making them cost-effective and suitable for qPCR systems for rapid mutation detection. This innovation is particularly beneficial for clinical and large-scale screening of specimens for specific point mutations, enhancing the efficiency and accuracy of molecular diagnosis.

## 2. Materials and Methods

### 2.1. Primer Design Concept

**Figure 1:**
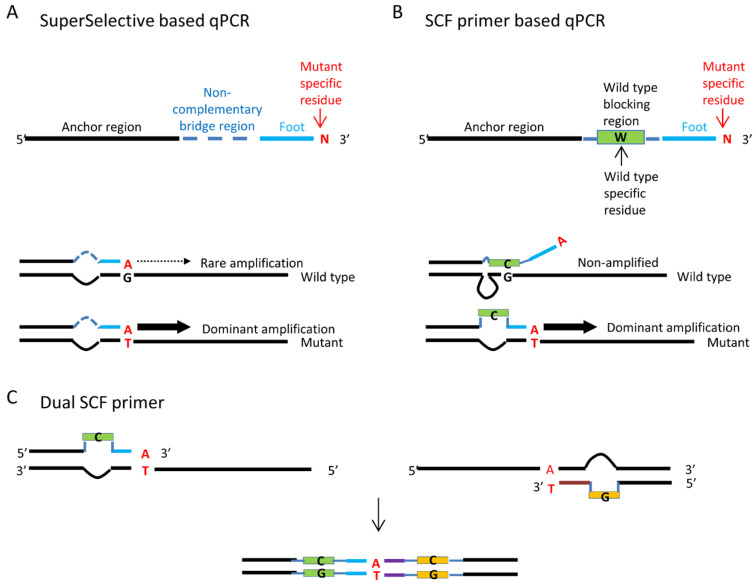
SCF Primer Design and Mechanism. (**A**) SuperSelective primer with anchor, non-complementary bridge, and foot regions. (**B**) SCF primer with wild-type blocking sequence in the bridge region to inhibit wild-type amplification. (**C**) Dual SCF primer approach for enhanced mutation detection. In the figure, the wild-type residuals are indicated in black letters, while the mutant residuals are indicated in red letters.

Superselective-based oligonucleotides consist of three regions from 5′ to 3′: the Anchor region, the non-complementary bridge region, and the Foot region. The Foot region sequence perfectly matches the target mutation site and its surrounding sequence (Figure 1A). To increase the specificity of SNV detection, SCF primers introduce a blocking sequence into the non-complementary bridge region of the original SuperSelective-based oligonucleotides. This blocking sequence matches the wild-type sequence of the target mutation site, thereby inhibiting non-specific PCR amplification that uses the wild-type sequence as a template (Figure 1B). The primer design followed specific parameters as initial design constraints prior to optimization: the total primer length ranged from 50–70 nucleotides, with an anchor region of 25–40 nucleotides (Tm 60–70 °C), a bridge region of 12–24 nucleotides that included 8–15 nucleotides of blocking sequence, and a mutation discrimination foot region of 6–12 nucleotides. To adapt to qPCR, the amplicon in each reaction was limited to <250 bp. The mutation-matching nucleotide was positioned at the 3′ end of the primer. Primers with different lengths of blocking sequence were synthesized to optimize wild-type blocking efficiency. For cases where a single SCF primer cannot achieve acceptable mutation discrimination, dual SCF primers for both directions of qPCR can be used to further enhance the ability to distinguish mutations (Figure 1C). The primer design for this study was assisted by the IDT Oligoanalyzer online Tool (Integrated DNA Technologies, Coralville, IA, USA; Available online: https://www.idtdna.com/pages/tools/oligoanalyzer (accessed on 13 January 2026). The primers were synthesized by Genomics (New Taipei city, Taiwan) and further purified by HPLC.

### 2.2. Ethics Statement

The study protocol was approved by the Institutional Review Board of MacKay Memorial Hospital (IRB number: 23MMHIS467e) and conducted in accordance with the Declaration of Helsinki (Version 2013).

### 2.3. Genomic DNA Extraction

Up to 1 g of tumor samples obtained from 86 endometrial cancer patients was chopped to 2–3 mm^3^ and resuspended in 4.7 mL of serum-free RPMI 1640. The chopped tissues were then transferred to a C-tube and prepared as single-cell suspensions using the Tumor Dissociation Kit (Miltenyi Biotec, Frankfurt am Main, Germany) in a gentleMACS Dissociator (Miltenyi Biotec, Frankfurt am Main, Germany) with built-in programs. The single-suspended cells were stored at −80 °C with CELLBANKER1 (Zenogen Pharma, Fukushima, Japan) in a concentration of 3~10 × 10^7^ cells/mL before further use. All genomic DNA from the single-cell suspended tumor samples was extracted using the QIAamp DNA Blood Mini Kit (Qiagen, Hilden, Germany) according to the manufacturer’s instructions. The concentration of extracted genomic DNA was determined using the Qubit dsDNA HS assay kit (Thermo Fisher Scientific, Waltham, MA, USA).

### 2.4. Establishment of POLE-EDM Carrier Stable Clones

For the sites of pathogenic POLE-EDMs that did not appear in NGS-analyzed endometrial cancer samples, including M295R and D368Y, the positive control mutations were established by genetic engineering through stable transfection of the mutant-containing fragments into the genomic DNA of 293FT cells. Briefly, the wild-type fragment covering M295 and D368 of the POLE gene was amplified from genomic DNA of 293FT cells by PCR with primers 5′-GGTTAGTCTTAGGGTCCTTC-3′ and 5′-GAAAGAGGACAGACAAGCAAG-3′. The PCR products were cloned using the T&A cloning kit (YB Biotech, New Taipei City, Taiwan) and further sequence-confirmed by Sanger sequencing. Site-specific mutagenesis was conducted using the Q5 Site-Directed Mutagenesis Kit (New England Biolabs, Ipswich, MA, USA) with primer pairs carrying specific mutation sites (For M295R, 5′-CCAGATTATGAG-GATTTCCTACATGATCGAT-3′ and 5′-TCTGTCTCAGCATCAGGAAACTTG-3′; for D368Y, 5′-GGACTTTTTTTACTGGTGAGTCTG-3′ and 5′-CCGTTGTAGGTGACC-3′). The mutated clones were sequence-confirmed again by Sanger sequencing. For mutant-containing stable clone generation, the DNA fragments of the POLE gene with specific mutations were subcloned into the pCDH-CMV-MCS-EF1α-Puro vector and transfected into 293FT cells using jetPRIME (Polyplus, Illkirch, France). The stable clones were selected with puromycin in DMEM medium containing 10% FBS (Thermo Fisher Scientific, Waltham, MA, USA) by limited dilution. For each selected clone, the ΔCt of qPCR was calculated by subtracting the β-actin Ct value from the target DNA fragment Ct value. The β-actin Ct was determined using primers 5′-TGCTGTGGAAGCTAAGTCCTG-3′ and 5′-GGAAAGACACCCACCTTGATCT-3′, while the target DNA fragment Ct was obtained using primers 5′-AACCAGAGGGAGGTAGAGCA-3′ and 5′-TCCCAGAAGCCACCTGCTCA-3′. All qPCR reactions were performed using 10 ng of genomic DNA extracted from each clone. The ΔCt was calculated by subtraction of β-actin Ct from the target DNA fragment Ct value of each clone, and the ΔΔCt of each clone was calculated by subtracting the ΔCt of 10 ng non-engineered 293FT genomic DNA from the ΔCt of each selected mutant carrying a stable clone. The copy number of the mutation integration fragment was calculated as follows:

Integration copy number = 2^(−ΔΔCt + 1) − 2

The frequency of mutation in the selected stable clone was further calculated as described below:

% of mutant = (2^(−ΔΔCt + 1) − 2)/2^(−ΔΔCt + 1) × 100%

### 2.5. qPCR Assay

To validate the effectiveness of SCF primers for SNV detection, the mutant-containing frequency of POLE-EDM-positive samples was determined based on reads of NGS for clinical samples or calculated from qPCR results as described above for established POLE-EDM stable clones and adjusted to 30% (10% for P436R mutant) by mixing with genomic DNA of 293FT cells as positive control for each site of mutant. Genomic DNA extracted from 293FT cells (Thermo Fisher, MA, USA) lacking POLE-EDM served as the wild-type control. The SuperSelective-based primers were designed to have the same sequence in the anchor and foot regions and the same length in the bridge region as SCF primers, to detect the same POLE-EDM sites. For individual qPCR reactions, we combined 10 ng of genomic DNA with a primer pair (1.25–5 pmole of each primer), 10 μL of FastStart Essential DNA Green Master (Roche, Basel, Switzerland), and an appropriate amount of TMAC (0~30 mM) to a final volume of 20 μL. To evaluate the quality of each sample, a primer pair specific for β-actin (5′-TGCTGTGGAAGCTAAGTCCTG-3′ and 5′-GGAAAGACACCCACCTTGATCT-3′) was used in the qPCR. The qPCR was performed using a LightCycler 96 (Roche, Switzerland) with the following program: one cycle at 95 °C for 10 min, followed by 40–50 cycles of 95 °C for 10 s and 62 °C for 30 s. An additional melting curve determination cycle was performed at the end of the qPCR. The qPCR results were analyzed using LightCycler 96 SW 1.1 software.

### 2.6. Statistics

Linear regression analysis was performed to evaluate the relationship between the logarithm (Log2) of mutation-containing frequencies and Ct values for each POLE-EDM site. Goodness-of-fit was assessed using the R^2^ value; R^2^ > 0.96 indicated a strong linear correlation. Statistical analyses were performed using Microsoft Excel 2007.

### 2.7. Next Generation Sequence for POLE EDM Detection

A targeted sequencing approach was employed for next-generation sequencing (NGS) to detect POLE exonuclease domain mutations (EDM). The NGS service was provided by the National Genomics Center for Clinical and Biotechnological Applications of the Cancer Progression Research Center (National Yang Ming Chiao Tung University, Taiwan). Briefly, the exon regions of the POLE gene covered by the probes are detailed in Table S1; these probes were designed and synthesized by QIAGEN. The sequencing process began with DNA fragmentation and library preparation using the TruSeq DNA PCR-Free HT Sample Prep Kit (Illumina, San Diego, CA, USA). The target regions were further enriched using SeqCap EZ target enrichment technology (Roche, Switzerland) with target-specific probes. Sequencing was then performed using the Illumina HiSeq system. The resulting sequence data was further analyzed using CLC BIO software (CLC Genomics Workbench 8.0, Qiagen, Hilden, Germany) to trim FASTQ files, map reads to reference genes, and generate a Variant Call Format (VCF) file. The variants were further annotated and filtered from VCF files using ANNOVAR (version 2013Aug23) to identify potential POLE mutations.

## 3. Results

### 3.1. SCF Primers Possess Superior Mutation Discrimination Ability

**Figure 2:**
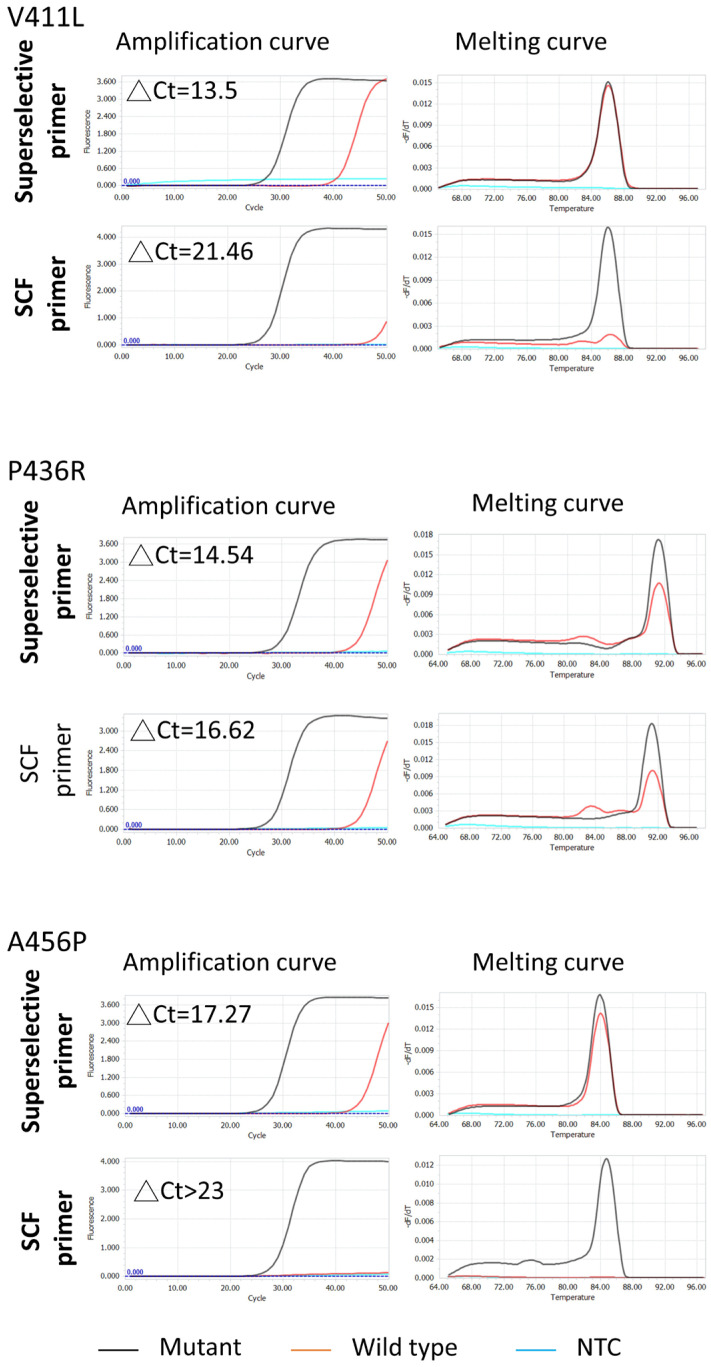
SCF vs. SuperSelective Primer Performance. qPCR amplification and melting curves for POLE V411L, P436R, and A456P mutations, showing higher ΔCt values for SCF primers compared to SuperSelective primers, indicating improved specificity. Genomic DNA (10 ng) from endometrial cancer tissues with mutations and 293FT cells (wild-type control) was used. ΔCt represents Ct_wildtype_-Ct_mutant_ in each experiment.

To evaluate the variant nucleotide detection ability of self-fishing primer-based qPCR, the endometrial cancer samples with POLE-EDMs confirmed by NGS were used as targets. Traditional SuperSelective primers with the same anchor and foot region sequences and the same bridge region length as SCF primers were designed and used in parallel as controls. The mutation-discrimination ability was determined by △the Ct values from qPCR results for the same sets of genomic DNA with and without specific mutations. The primer concentration and the recipes of qPCR reagents were identical in each pair of comparisons for POLE-EDM detection. Figure 2 shows that SCF primer-based qPCR, in the examples of POLE-V411L, P436R, and A456P mutation detection, possessed higher ΔCt than traditional SuperSelective primer-based qPCR control between the same set of variant and non-variant templates. These results demonstrated that the SCF primer-based qPCR strategy can improve the mutation-distinguishing ability of the traditional SuperSelective primer-based qPCR for clinical applications.

### 3.2. Alter the Blocking Sequence Length and Cover Region to Optimize the SNV Discrimination Ability of SCF Primers

**Figure 3:**
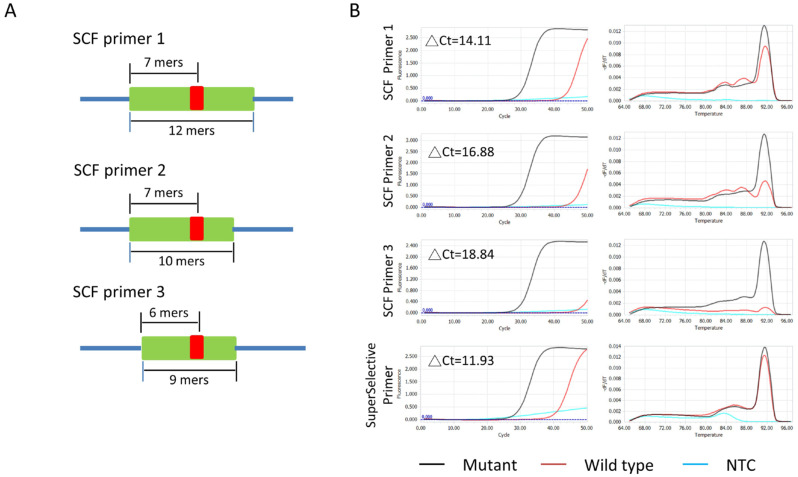
Optimization of SCF Blocking Sequence. (**A**) SCF primer designs for POLE-M444K with blocking sequence lengths of 9–12 nucleotides and distances of 6–7 nucleotides from the mutation site (green: blocking sequence; red: mutation site). (**B**) qPCR results showing higher ΔCt values with optimized SCF primers compared to SuperSelective primers, using 10 ng of POLE-M444K mutant DNA and 293FT wild-type DNA. ΔCt represents Ct_wildtype_-Ct_mutant_ in each experiment.

The superior SNV-discrimination ability of SCF primers is due to the inclusion of a blocking sequence in the bridge region. However, the criteria for the blocking sequence design are unclear. To understand this problem, we designed blocking sequences with different parameters, including the length of the blocking sequence and the distance from its 3′ end to the variation point. The POLE-M444K was represented as an example. The total lengths of the blocking sequence were designed from 9~12 mers, and the distance from the 5′ end of the blocking sequence to the variant point was 6~7 mers (Figure 3A). By testing with the same sets of POLE-M444K carrier genomic DNA extracted from an endometrial cancer sample and wild-type genomic DNA extracted from 293FT cells, the primer without adding a blocking sequence (the SuperSelective primer) possesses the smallest ΔCt value after qPCR reaction and demonstrates the poorest SNV discrimination ability. However, all SCF primer designs had higher ΔCt values than the Superselective design, and altering the parameter described above can alter SNV discrimination ability (Figure 3B). The longer length of the blocking sequence and the distance from its 5′ end to the variant point led to non-specific amplification, as indicated by the melting curves. These results demonstrated that the SNV-discrimination ability of the self-competitive primer can be optimized by altering the cover region of the blocking sequence.

### 3.3. Dual SCF Primer qPCR Improved the SNV Distinguishability

**Figure 4:**
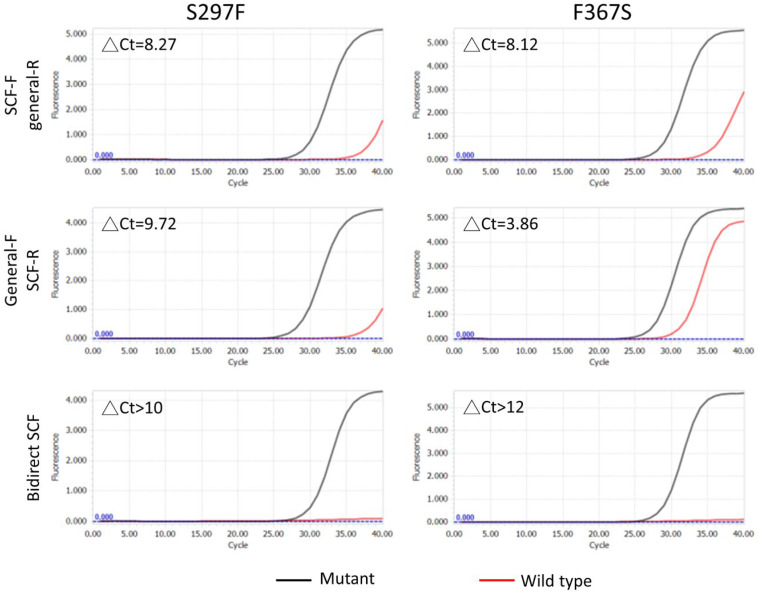
Dual SCF Primer Enhancement. qPCR results for POLE S297F and F367S, comparing single SCF primers (forward SCF with general reverse, or general forward with SCF reverse) with dual SCF primers (bidirectional SCF), showing superior discrimination with dual primers using 10 ng of mutant and wild-type DNA (293FT cells as wild-type control). All reactions were conducted under the same conditions. ΔCt represents Ct_wildtype_-Ct_mutant_ in each experiment.

In contexts where qPCR with a single SCF cannot achieve satisfactory SNV discrimination, regardless of primer orientation, we applied dual SCF primer qPCR to overcome this limitation. By utilizing SCF primer pairs from both forward and reverse directions, the ΔCt values of genomic DNA with 30% of S297F and F367S mutation subtracted from wild-type genomic DNA were higher than those achieved with single-direction SCF primers with the identical sequences (Figure 4). These results demonstrate that dual SCF primer qPCR exhibits superior SNV-distinguishing ability when single-competitive fishing primers fail to yield satisfactory results. This dual SCF primer system can be used effectively when the single SCF primer qPCR system fails to provide reliable results.

### 3.4. Sensitivity of POLE-EDM Detection with SCF Primer-Based qPCR

**Figure 5:**
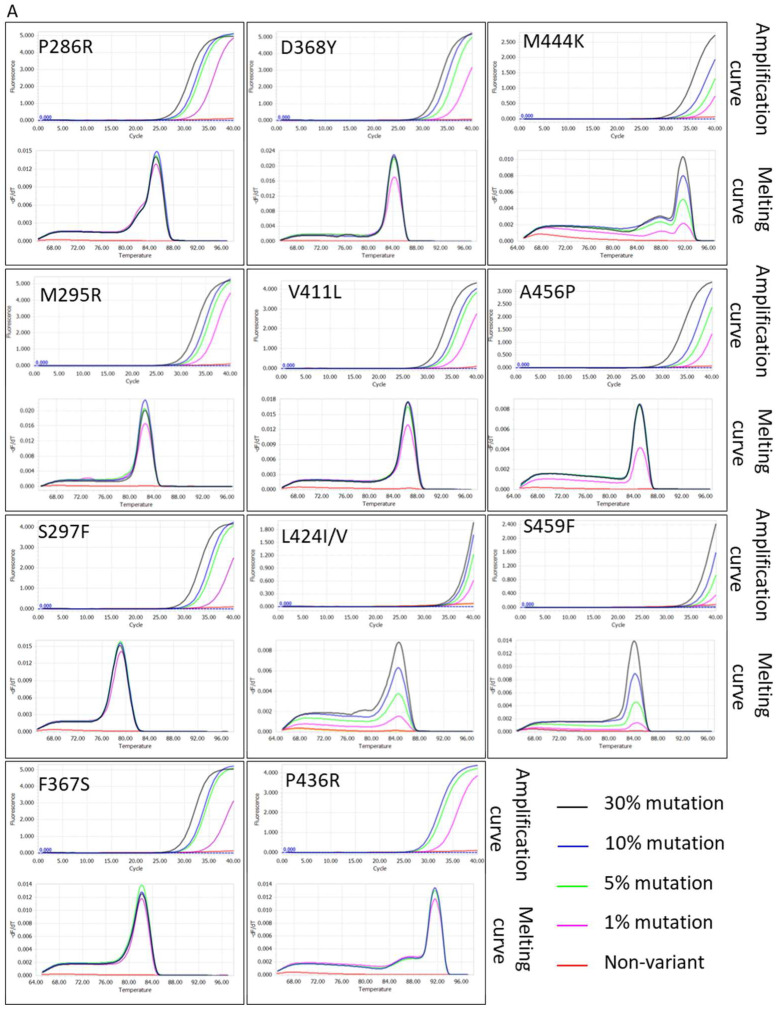

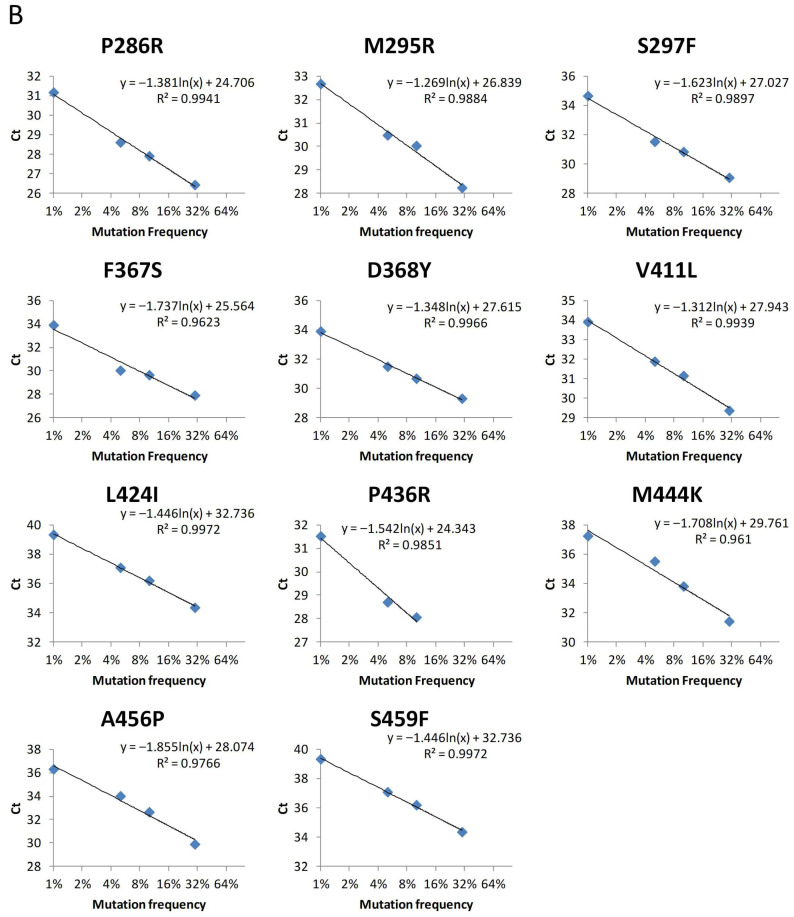
Sensitivity of SCF qPCR. (**A**) Amplification and melting curves for POLE EDMs at mutation frequencies from 1% to 30%, with melting curves confirming specificity. Genomic DNA (10 ng) from endometrial cancer samples was diluted with 293FT wild-type DNA. (**B**) Linear regression of Ct values vs. log2 mutation frequencies (R^2^ > 0.96), calculated using Microsoft Excel.

A tumor lesion comprises not only cancer cells but also stromal cells and various immune cells. Additionally, tumor heterogeneity influences the ratio of cancer cells with specific sequence variants. Therefore, the tumor cells with particular mutations in a single clinical specimen are often rare. To detect these mutations, high sensitivity is required. Eleven pathogenic mutations located in the POLE exonuclease domain were selected as a panel in the test (Table 1). Among them, P286R, S297F, V411L-T/C, A456P, and S459F occupied over 95% of mutations, especially P286R and V411L-T/C. Minor variations occurred at sites including M295R, F367S, D368Y, L424I/V, and M444K, accounting for approximately 4.4% of all variations [20,21]. Thus, detection of pathogenic POLE-EDMs by using SCF primer-based qPCR was focused on these 11 sites. By serially diluting clinical sample genomic DNA containing specific POLE-EDMs (the mutation sites and mutation frequencies were determined by NGS) with genomic DNA from 293FT cells to 10 ng final, the sensitivity of POLE-EDM detection using SCF primer-based qPCR with optimized primer sequences can reach 1% (~15 copies in samples), with all positive signals appearing at CT values < 40 (Figure 5A). The R-square values of the linear regression curve between Log2 of mutation-containing frequencies and Ct values were >0.96 in all pathogenic POLE-EDM points (Figure 5B). These results demonstrated that the SCF primer-based qPCR system has sufficiently high SNV detection sensitivity and is suitable for POLE-EDM tumor detection in clinical settings.

### 3.5. Interpretation of POLE-EDM Detection Results by Using SCF Primer-Based qPCR

**Figure 6:**
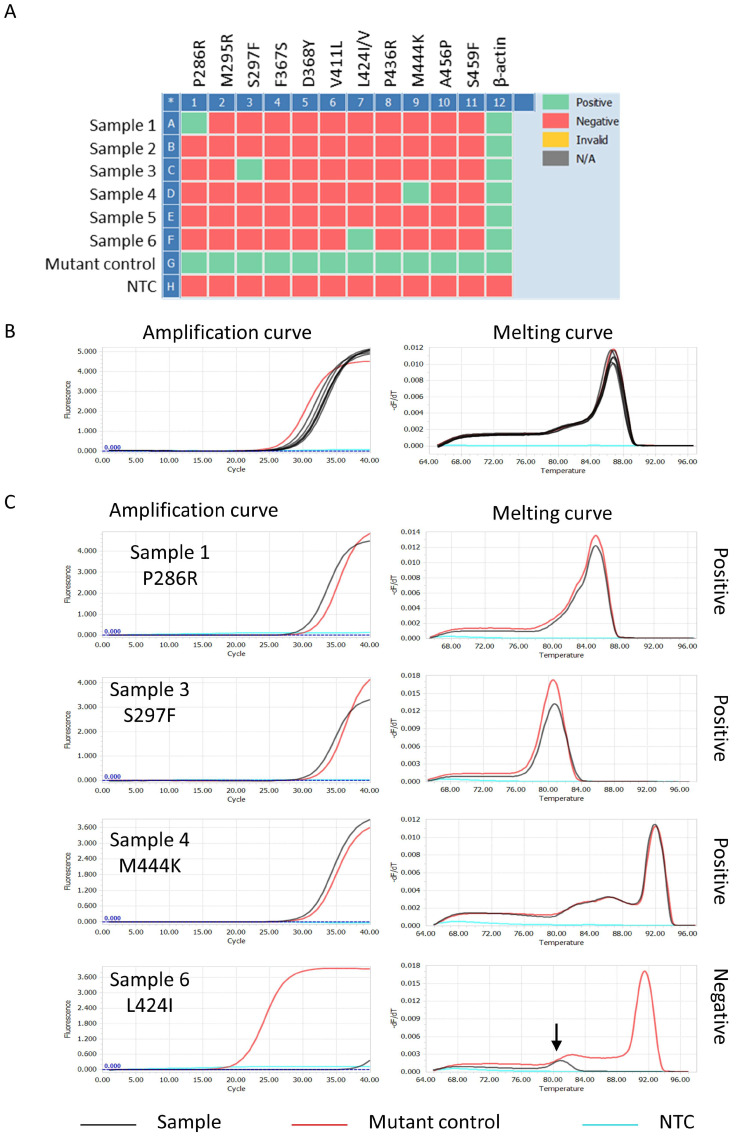
POLE-EDM Detection Workflow (**A**) qPCR setup for 6 samples, positive controls, and non-template controls (green: positive signals; red: negative signals) (Asterisks (*) indicate software-generated row/column separators and have no specific meaning). (**B**) β-actin qPCR for sample quality control. Multiple lines of the same color indicate the amplification and melting curves derived from each individual testing sample. (**C**) Mutation identification by comparing melting curves with positive controls for samples 1 (P286R), 3 (S297F), 4 (M444K), and 6 (L424I); overlapping curves indicate mutations. The arrow indicates a melting curve that does not match the mutant control.

Since amplification signals from wild-type 293FT genomic DNA at all loci appeared at CT > 40, the qPCR assay was set to run for 40 cycles to simplify result interpretation during sample testing. The reaction performed in the 96-well reaction plate was designed for 6 samples with a set of positive controls and a non-template control for each mutation allele, as shown in Figure 6A. The qPCR results for the β-actin gene were first evaluated to assess quality and sample loading control (Figure 6B). Mutation alleles with amplified signals in each sample were selected, and their melting curves were further compared with the positive control group. Those that overlap entirely were determined to carry the mutation at that site, while those that did not overlap were still determined not to have that mutation (Figure 6C). Based on the analysis design, the mutation status of these 11 POLE-EDM loci can be examined in a single step, yielding results without complex analysis.

### 3.6. Validated the SCF Primer-Based qPCR Results with NGS

To further validate the precision of the SCF primer-based qPCR results, genomic DNA extracted from 86 endometrial cancer tissues was analyzed using both SCF primer-based qPCR and panel-based exon capture NGS for POLE-EDM detection. For reproducibility, the qPCR analysis was conducted by multiple technicians using the same qPCR instrument (LightCycler 96, Roche), blinded to NGS results. The association between 11 pathogenic POLE-EDMs detected by NGS and the clinical outcome of these endometrial cancer subjects is presented in Figure S1. The variants detected at these 11 loci using the self-fishing primer qPCR method were compared with the NGS results for each sample. The results were in complete agreement (Table 2 and Table S2), demonstrating that this method can accurately, rapidly, and economically identify the variant status of these 11 POLE-EDM loci in samples.

## 4. Discussion

With the increasing demand and advancements in precision medicine, the clinical importance of specific genetic mutations has been gradually elucidated. Big data statistics have facilitated the identification of optimal treatment strategies for patients with these mutations, particularly in malignant tumors [22]. Although Next-Generation Sequencing (NGS) strategies provide a comprehensive understanding of cancer cell genetic mutations, the high analysis costs, time to report, sequencing error rates, result interpretation, and analytical sensitivity constraints continue to limit their widespread clinical application [23]. Nevertheless, the extensive use of NGS as a research tool has enabled the identification of the most crucial genetic mutations for molecular classification by comparing results with tumor prognoses [8]. This has significantly reduced the number of genetic mutations that need to be monitored in specific tumors, making it feasible to use non-NGS strategies for analysis, thereby better meeting clinical needs in terms of cost and time.

This study developed an SCF primer-based qPCR strategy to detect multiple POLE-EDMs in endometrial cancer. This method enables rapid and low-tech analysis in general molecular diagnostic laboratories. Compared to the original DPO primers, incorporating a self-competitive oligonucleotide fragment within the primers effectively enhances the ability to distinguish mutation sites and reduces false positives. Moreover, unlike other WTB or self-competitive primer methods, this design does not require specially modified oligonucleotides (such as Locked Nucleic Acid) or additional Sanger sequencing for result interpretation, making it more cost-effective. However, this method relies on short 3′ fragments to determine specific mutations, so a thorough understanding of post-mutation sequences is necessary for its application. It has limitations in detecting uncertain variations (such as insertions/deletions or closely spaced multiple mutations), which may interfere with the binding affinity of the foot region and blocking region of primers to target templates, thereby affecting the accuracy of target mutation point detection. Nevertheless, established pathogenic POLE-EDMs sequence variations are fixed, and all are single-nucleotide alterations. Given the extremely low frequency of mutations other than P286R/V411L, the probability of multiple mutations occurring simultaneously is negligible, making this method suitable for detecting pathogenic POLE-EDMs. While the current SCF design works best for single-nucleotide variations, future adaptations with more flexible primer structures may extend its application to more complex mutation types.

In general, the longer the length of the blocking sequence, the better the inhibition effect for mismatch amplification of wild-type targets. However, it may also cause non-specific amplification of off-target regions by over-restricting amplification in the target region. On the other hand, the competition between the blocking sequence and the foot region of SCF primers is determined by the upstream region of the SNV to be distinguished. The sequence around the variant site further complicates the situation; therefore, attempting different lengths of blocking sequences and varying the distance between the blocking sequence’s 5′ end and the variant site helps identify the optimal SCF primer sequence for SNV discrimination.

It appears contradictory that SCF primer pairs can improve SNV distinguishing ability because the products that self-fishing primers amplified carried target mutations that might be used as templates for reverse direct pairing SCF primers, regardless of whether the original templates contained mutations or not. The possible explanation is that SCF primers tend to amplify the target variant template more than the wild-type template, thereby producing a synergistic effect when dual SCF primers are used for mutation detection. The same strategy had been applied to SuperSelective primer pairs for rare somatic KRAS and EGFR mutation detection, with superior SNV-distinguishing ability compared to qPCR using a single SuperSelective primer and a conventional reverse primer [24].

Pathogenic POLE mutation detection has been reported using quantitative PCR with particular fluorescent probes targeting mutant or wild-type sequences of each detection allele [25]. The results of mutation detection are determined by analyzing plots of wild-type and variant-specific qPCR quantities in a two-dimensional scatter plot, an approach for detecting specific mutations in tumor specimens. This approach can also yield results quickly and cost-effectively. However, a few ambiguous results require additional effort to confirm and establish appropriate cutoff criteria. Therefore, restricting the amplification power to achieve clinically acceptable sensitivity is needed to simplify this process. In the present study, by using fixed amounts of sample nucleic acid and setting qPCR amplification cycles based on a 1% detection threshold, we avoided obtaining uncertain results. Of course, analyzing more samples and different sample sources (such as paraffin-embedded tissues) from which nucleic acids are extracted is indeed necessary to confirm these findings. Because the amplicons generated by SCF qPCR in this study were <250 bps, they fall within the typical fragment size range observed in FFPE-derived DNA (100–400 bp in moderately degraded samples). This suggests that the assay would be applicable to such specimens, although further validation is required.

Due to NGS bias and qPCR performance variations across different target sequences, there may be errors in the proportions of mutation sites in the reference standards used in this study, particularly when clinical samples with POLE-EDM or generated POLE-EDM carrier stable clones are used. To correct this situation, a knock-in POLE-EDM in situ using CRISPR technology can generate reliable reference standards for further application [26].

The accuracy of primer sequences is essential for this detection system. Poor primer quality can significantly affect the detection results. In addition to proper primer storage, high-quality primer purification, such as using HPLC, is also required. Furthermore, primers synthesized with phosphorothioate (PS) bond-modified nucleotides are recommended to prevent exonuclease degradation [27].

Other factors to consider include the differences in sensitivity between the SCF primer and other mutation detection methods, and their impact on results. The sensitivity of NGS as a mutation-detection method is often affected by read depth and data filtering, leading to false-negative results [28]. Therefore, using it as validation data to evaluate the accuracy of the SCF primer system remains debatable. Other methods, such as digital PCR, might be more suitable alternatives [29].

Because of the complexity in operation and requirement for different facilities, the benefit of the SCF-qPCR for POLE-EDM extends beyond cost and time. Table 3 summarizes the advantages of SCF qPCR over NGS-based methods for POLE-EDM detection. The availability of the required instrumentation and the simplicity of the assay procedure further demonstrate that SCF qPCR is more feasible for routine clinical practice.

## 5. Conclusions

In conclusion, this study developed a self-competitive primer-based qPCR method with enhanced specificity for detecting 11 major POLE-EDM sites in a cost-effective and efficient manner. This method may facilitate complete molecular classification of endometrial cancer and has strong potential for broad clinical application.

## Figures and Tables

**Table 1:** Pathogenic POLE-EDM Detection Panel.

Amino Acid Substitution	Nucleotide Substitution
P286R	c.857C>G
M295R	c.884T>G
S297F	c.890C>T
F367S	c.1100T>C
D368Y	c.1102G>T
V411L	c.1231G>T/C
L424I/V	c.1270C>A/G
P436R	c.1307C>G
M444K	c.1331T>A
A456P	c.1366G>C
S459F	c.1376C>T

Lists 11 pathogenic POLE EDMs targeted by the SCF qPCR system, including amino acid and nucleotide substitutions (e.g., P286R, c.857C>G) covering >95% of reported pathogenic EDM.

**Table 2:** Comparison of SCF qPCR and NGS Results (N = 86).

Mutation	qPCR	NGS
P286R	4	4
M295R	0	0
S297F	1	1
F367S	1	1
D368Y	0	0
V411L	4	4
L424I/V	1	1
P436R	1	1
M444K	1	1
A456P	1	1
S459F	1	1
Negative *	71	71
Total	86	

Summarizes the concordance between SCF qPCR and NGS for detecting POLE EDMs in 86 endometrial cancer samples, showing identical variant detection. * Negative means that no mutation was detected in the listed 11 loci.

**Table 3:** The advantage of SCF qPCR for POLE-EDM detection.

Item	SCF qPCR	NGS
Cost	~$15 USD/sample	$1300~4000 USD/sample
Time	4–6 h	several weeks
Facility	general qPCR	professional NGS equipment
Operation	easy	complex

## Data Availability

The original contributions presented in this study are included in the article/Supplementary Material. Further inquiries can be directed to the corresponding author.

## Supplementary Materials

The following supporting information can be downloaded at: https://www.mdpi.com/article/10.3390/cimb48030257/s1.

## Abbreviations

The following abbreviations are used in this manuscript:

SCF	Self-Competitive Fishing
EDMs	Exonuclease domain mutations
SNV	Single-nucleotide variant
NGS	Next-generation sequencing
