# Supplementary material for: A Novel Self-Competitive Fishing Primer qPCR Approach for Efficient POLE Mutation Detection in Endometrial Cancer Molecular Classification
Source: Curr Issues Mol Biol. 2026 Feb 27;48(3):257. doi: 10.3390/cimb48030257 (PMC13025916; doi:10.3390/cimb48030257)
Supplement: Supplementary file 1 [file cimb-48-00257-s001.zip › Supplementary Figure 1.docx]

**
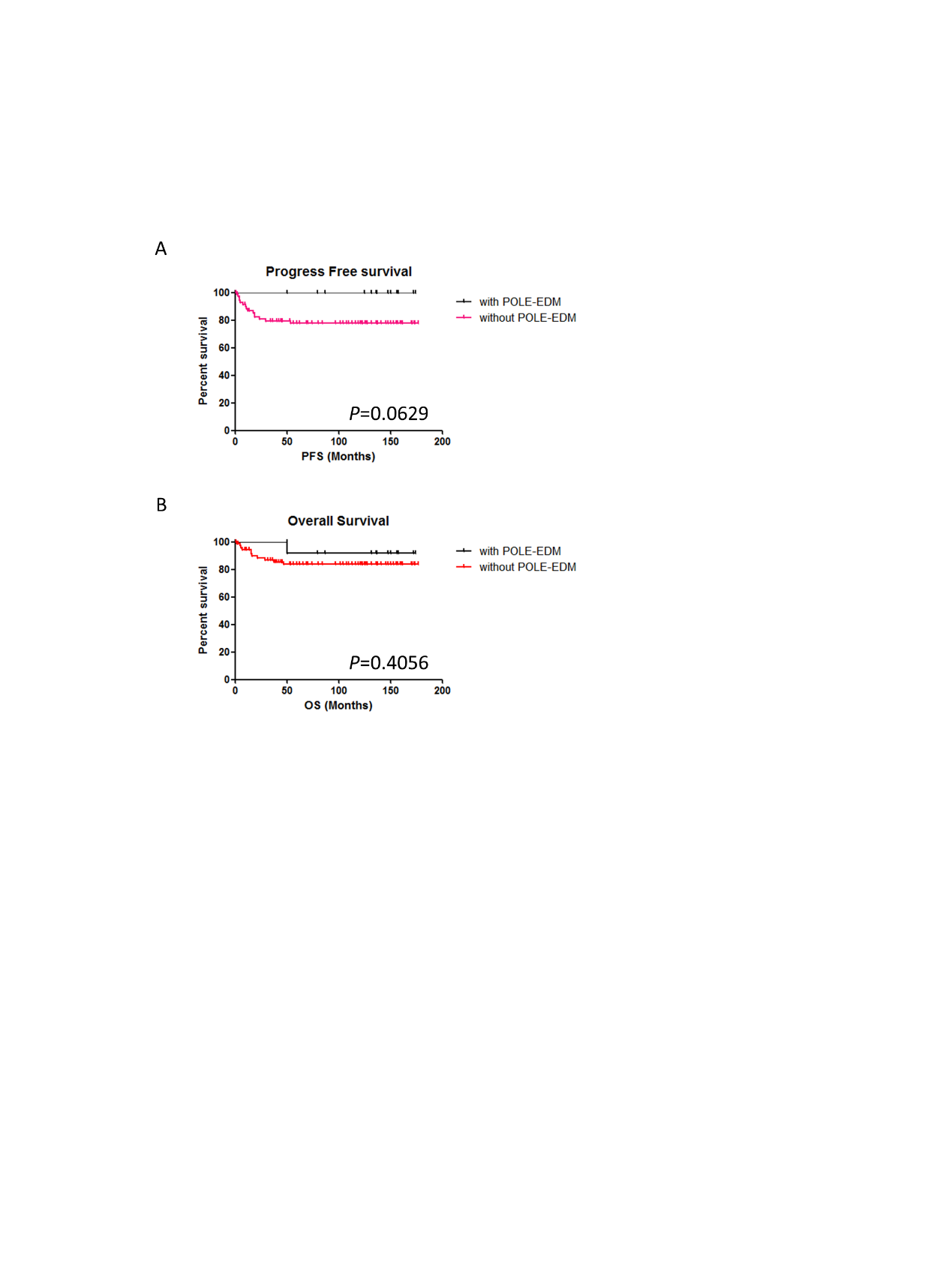
**

**Figure S1. Survival Analysis by POLE-EDM Status**

Kaplan-Meier curves for (A) progression-free survival (P=0.4056) and (B) overall survival (P=0.0629) stratified by POLE-EDM status, determined by next-generation sequencing (N=86).
