# Supplementary material for: A Novel Self-Competitive Fishing Primer qPCR Approach for Efficient POLE Mutation Detection in Endometrial Cancer Molecular Classification
Source: Curr Issues Mol Biol. 2026 Feb 27;48(3):257. doi: 10.3390/cimb48030257 (PMC13025916; doi:10.3390/cimb48030257)
Supplement: Supplementary file 1 [file cimb-48-00257-s001.zip › Supplementary Table 1.pdf]

Table S1. POLE Exon Capture Regions for NGS

Details chromosome regions (based on GRCh37/hg19) targeted for NGS analysis of POLE EDMs.

Gene	Chromosome	start	end
POLE	chr12	133201282	133201396
		133201490	133201580
		133202230	133202356
		133202702	133202903
		133208900	133209094
		133209249	133209381
		133210771	133210964
		133212477	133212610
		133214599	133214725
		133215710	133215884
		133218232	133218437
		133218762	133218983
		133219091	133219315
		133219405	133219582
		133219809	133219916
		133219992	133220146
		133220422	133220563
		133225514	133225658
		133225891	133226101
		133226262	133226475
		133233721	133233844
		133233934	133234015
		133234453	133235260
		133235880	133236095
		133237554	133237750
		133238112	133238270
		133240589	133240734
		133240955	133241048
		133241887	133242036
		133244088	133244234

		133244941	133245088
		133245220	133245323
		133245396	133245525
		133248800	133248908
		133249212	133249425
		133249749	133249863
		133250160	133250293
		133251983	133252103
		133252320	133252406
		133252679	133252770
		133253131	133253239
		133253948	133254029
		133254163	133254305
		133256082	133256237
		133256539	133256632
		133256763	133256808
		133257192	133257273
		133257723	133257865
		133263839	133263901
