# Supplementary material for: A Novel Self-Competitive Fishing Primer qPCR Approach for Efficient POLE Mutation Detection in Endometrial Cancer Molecular Classification
Source: Curr Issues Mol Biol. 2026 Feb 27;48(3):257. doi: 10.3390/cimb48030257 (PMC13025916; doi:10.3390/cimb48030257)
Supplement: Supplementary file 1 [file cimb-48-00257-s001.zip › Supplementary Table 1.docx]

**Table S1. POLE Exon Capture Regions for NGS**Details chromosome regions (based on GRCh37/hg19) targeted for NGS analysis of POLE EDMs.

| Gene | Chromosome | start | end |
| --- | --- | --- | --- |
| POLE | chr12 | 133201282 | 133201396 |
|  |  | 133201490 | 133201580 |
|  |  | 133202230 | 133202356 |
|  |  | 133202702 | 133202903 |
|  |  | 133208900 | 133209094 |
|  |  | 133209249 | 133209381 |
|  |  | 133210771 | 133210964 |
|  |  | 133212477 | 133212610 |
|  |  | 133214599 | 133214725 |
|  |  | 133215710 | 133215884 |
|  |  | 133218232 | 133218437 |
|  |  | 133218762 | 133218983 |
|  |  | 133219091 | 133219315 |
|  |  | 133219405 | 133219582 |
|  |  | 133219809 | 133219916 |
|  |  | 133219992 | 133220146 |
|  |  | 133220422 | 133220563 |
|  |  | 133225514 | 133225658 |
|  |  | 133225891 | 133226101 |
|  |  | 133226262 | 133226475 |
|  |  | 133233721 | 133233844 |
|  |  | 133233934 | 133234015 |
|  |  | 133234453 | 133235260 |
|  |  | 133235880 | 133236095 |
|  |  | 133237554 | 133237750 |
|  |  | 133238112 | 133238270 |
|  |  | 133240589 | 133240734 |
|  |  | 133240955 | 133241048 |
|  |  | 133241887 | 133242036 |
|  |  | 133244088 | 133244234 |
|  |  | 133244941 | 133245088 |
|  |  | 133245220 | 133245323 |
|  |  | 133245396 | 133245525 |
|  |  | 133248800 | 133248908 |
|  |  | 133249212 | 133249425 |
|  |  | 133249749 | 133249863 |
|  |  | 133250160 | 133250293 |
|  |  | 133251983 | 133252103 |
|  |  | 133252320 | 133252406 |
|  |  | 133252679 | 133252770 |
|  |  | 133253131 | 133253239 |
|  |  | 133253948 | 133254029 |
|  |  | 133254163 | 133254305 |
|  |  | 133256082 | 133256237 |
|  |  | 133256539 | 133256632 |
|  |  | 133256763 | 133256808 |
|  |  | 133257192 | 133257273 |
|  |  | 133257723 | 133257865 |
|  |  | 133263839 | 133263901 |
