# Supplementary material for: A Novel Self-Competitive Fishing Primer qPCR Approach for Efficient POLE Mutation Detection in Endometrial Cancer Molecular Classification
Source: Curr Issues Mol Biol. 2026 Feb 27;48(3):257. doi: 10.3390/cimb48030257 (PMC13025916; doi:10.3390/cimb48030257)
Supplement: Supplementary file 1 [file cimb-48-00257-s001.zip › Supplementary Table 2.pdf]

Table S2. Pathogenic POLE-EDM Variants in 86 Samples

Provides a detailed comparison of SCF qPCR and NGS results for each sample, with non-variant cases marked as "n.v."

Sample list	Self-competitive Fishing qPCR	NGS
		Mutant (mutant reads/total reads)
EMS012	A456P	A456P c.1366G>C (355/813)
EMS013	n.v.	n.v.
EMS015	n.v	n.v
EMS022	P286R	P286R c.857C>G (533/1641)
EMS028	n.v	n.v
EMS030	n.v	n.v
EMS031	n.v	n.v
EMS033	P286R	P286R c.857C>G (269/1786)
EMS034	n.v	n.v
EMS035	n.v	n.v
EMS036	n.v	n.v
EMS038	n.v	n.v
EMS040	n.v	n.v
EMS041	n.v	n.v
EMS042	V411L	V411L c.1231G>C (52/483)
EMS043	n.v	n.v
EMS044	n.v	n.v
EMS046	n.v	n.v
EMS047	n.v	n.v
EMS048	n.v	n.v
EMS049	n.v	n.v
EMS050	n.v	n.v
EMS051	n.v	n.v
EMS052	n.v	n.v
EMS053	n.v	n.v
EMS054	n.v	n.v
EMS055	n.v	n.v
EMS056	n.v	n.v

EMS057	n.v	n.v
EMS058	F367S	F367S T>C c.1100(304/2626)
EMS059	n.v	n.v
EMS060	P436R	P436R c.1307C>G (153/1466)
EMS062	n.v	n.v
EMS063	n.v	n.v
EMS064	n.v	n.v
EMS065	P286R	P286R c.857C>G (879/2780)
EMS066	n.v	n.v
EMS068	n.v	n.v
EMS069	n.v	n.v
EMS070	n.v	n.v
EMS071	n.v	n.v
EMS072	S297F	S297F c.890C>T (367/1216)
EMS073	n.v	n.v
EMS074	n.v	n.v
EMS075	n.v	n.v
EMS076	P286R	P286R C>G c.857(364/1279)
EMS077	n.v	n.v
EMS078	n.v	n.v
EMS080	n.v	n.v
EMS081	n.v	n.v
EMS082	n.v	n.v
EMS083	M444K	M444K c.1331T>A (273/681)
EMS084	n.v	n.v
EMS086	n.v	n.v
EMS087	n.v	n.v
EMS088	n.v	n.v
EMS089	V411L	V411L c.1231G>T (398/918)
EMS090	n.v	n.v
EMS091	n.v	n.v
EMS092	n.v	n.v
EMS093	V411L	V411L c.1231G>T (240/696)
EMS094	n.v	n.v
EMS096	n.v	n.v
EMS097	L424I/V	L424V c.1270C>G (520/1577)
EMS099	n.v	n.v

EMS100	n.v	n.v
EMS101	n.v	n.v
EMS102	n.v	n.v
EMS103	V411L	V411L c.1231G>C (5/440)
EMS104	n.v	n.v
EMS105	n.v	n.v
EMS106	n.v	n.v
EMS107	n.v	n.v
EMS108	n.v	n.v
EMS109	n.v	n.v
EMS111	n.v	n.v
EMS112	n.v	n.v
EMS113	S459F	S459F c.1376C>T (87/458)
EMS116	n.v	n.v
EMS119	n.v	n.v
EMS120	n.v	n.v
EMS122	n.v	n.v
EMS124	n.v	n.v
EMS125	n.v	n.v
EMS128	n.v	n.v
EMS130	n.v	n.v
