# Supplementary material for: A Novel Self-Competitive Fishing Primer qPCR Approach for Efficient POLE Mutation Detection in Endometrial Cancer Molecular Classification
Source: Curr Issues Mol Biol. 2026 Feb 27;48(3):257. doi: 10.3390/cimb48030257 (PMC13025916; doi:10.3390/cimb48030257)
Supplement: Supplementary file 1 [file cimb-48-00257-s001.zip › Supplementary Table 2.docx]

**Table S2. Pathogenic POLE-EDM Variants in 86 Samples**Provides a detailed comparison of SCF qPCR and NGS results for each sample, with non-variant cases marked as “n.v.”

| Sample list | Self-competitive Fishing qPCR | NGS |
| --- | --- | --- |
|  |  | Mutant (mutant reads/total reads) |
| EMS012 | A456P | A456P c.1366G>C (355/813) |
| EMS013 | n.v. | n.v. |
| EMS015 | n.v | n.v |
| EMS022 | P286R | P286R c.857C>G (533/1641) |
| EMS028 | n.v | n.v |
| EMS030 | n.v | n.v |
| EMS031 | n.v | n.v |
| EMS033 | P286R | P286R c.857C>G (269/1786) |
| EMS034 | n.v | n.v |
| EMS035 | n.v | n.v |
| EMS036 | n.v | n.v |
| EMS038 | n.v | n.v |
| EMS040 | n.v | n.v |
| EMS041 | n.v | n.v |
| EMS042 | V411L | V411L c.1231G>C (52/483) |
| EMS043 | n.v | n.v |
| EMS044 | n.v | n.v |
| EMS046 | n.v | n.v |
| EMS047 | n.v | n.v |
| EMS048 | n.v | n.v |
| EMS049 | n.v | n.v |
| EMS050 | n.v | n.v |
| EMS051 | n.v | n.v |
| EMS052 | n.v | n.v |
| EMS053 | n.v | n.v |
| EMS054 | n.v | n.v |
| EMS055 | n.v | n.v |
| EMS056 | n.v | n.v |
| EMS057 | n.v | n.v |
| EMS058 | F367S | F367S T>C c.1100(304/2626) |
| EMS059 | n.v | n.v |
| EMS060 | P436R | P436R c.1307C>G (153/1466) |
| EMS062 | n.v | n.v |
| EMS063 | n.v | n.v |
| EMS064 | n.v | n.v |
| EMS065 | P286R | P286R c.857C>G (879/2780) |
| EMS066 | n.v | n.v |
| EMS068 | n.v | n.v |
| EMS069 | n.v | n.v |
| EMS070 | n.v | n.v |
| EMS071 | n.v | n.v |
| EMS072 | S297F | S297F c.890C>T (367/1216) |
| EMS073 | n.v | n.v |
| EMS074 | n.v | n.v |
| EMS075 | n.v | n.v |
| EMS076 | P286R | P286R C>G c.857(364/1279) |
| EMS077 | n.v | n.v |
| EMS078 | n.v | n.v |
| EMS080 | n.v | n.v |
| EMS081 | n.v | n.v |
| EMS082 | n.v | n.v |
| EMS083 | M444K | M444K c.1331T>A (273/681) |
| EMS084 | n.v | n.v |
| EMS086 | n.v | n.v |
| EMS087 | n.v | n.v |
| EMS088 | n.v | n.v |
| EMS089 | V411L | V411L c.1231G>T (398/918) |
| EMS090 | n.v | n.v |
| EMS091 | n.v | n.v |
| EMS092 | n.v | n.v |
| EMS093 | V411L | V411L c.1231G>T (240/696) |
| EMS094 | n.v | n.v |
| EMS096 | n.v | n.v |
| EMS097 | L424I/V | L424V c.1270C>G (520/1577) |
| EMS099 | n.v | n.v |
| EMS100 | n.v | n.v |
| EMS101 | n.v | n.v |
| EMS102 | n.v | n.v |
| EMS103 | V411L | V411L c.1231G>C (5/440) |
| EMS104 | n.v | n.v |
| EMS105 | n.v | n.v |
| EMS106 | n.v | n.v |
| EMS107 | n.v | n.v |
| EMS108 | n.v | n.v |
| EMS109 | n.v | n.v |
| EMS111 | n.v | n.v |
| EMS112 | n.v | n.v |
| EMS113 | S459F | S459F c.1376C>T (87/458) |
| EMS116 | n.v | n.v |
| EMS119 | n.v | n.v |
| EMS120 | n.v | n.v |
| EMS122 | n.v | n.v |
| EMS124 | n.v | n.v |
| EMS125 | n.v | n.v |
| EMS128 | n.v | n.v |
| EMS130 | n.v | n.v |
